# Urinary detection of high-risk HPV DNA to enhance cervical cancer screening in developing countries

**DOI:** 10.1128/spectrum.01938-24

**Published:** 2025-07-18

**Authors:** Novia Syari Intan, Revata Utama, Dewi Wulandari, Reiva Wisdharilla, Shafira Mutia Khanza, Muhamad Rifki Ramadhan, Indah Suci Widyahening, Neni Nurainy, Rini Mulia Sari, Andrijono

**Affiliations:** 1 Nusantics, PT Riset Nusantara Genetika, East Jakarta, Indonesia; 2 Department of Clinical Pathology, Faculty of Medicine, Universitas Indonesia 95338 , Central Jakarta, Indonesia; 3 Cipto Mangunkusumo Hospital 364090 , Central Jakarta, Indonesia; 4 Department of Community Medicine, Faculty of Medicine, Universitas Indonesia 95338 , Central Jakarta, Indonesia; 5 Translational Development of Life Science Product Division, PT Bio Farma 294016 , Bandung, Indonesia; 6 Surveillance and Clinical Research Division, PT Bio Farma 294016 , Bandung, Indonesia; Emory University School of Medicine, Atlanta, Georgia, USA

**Keywords:** cervical cancer, early diagnosis of cancer, HPV, human papillomavirus, HPV DNA test, *in vitro* diagnostic medical devices, real-time PCR, multiplex PCR, self-sampling, urine, PathoScan hrHPV

## Abstract

To increase cervical cancer screening capacity and participation, we evaluated the performance of the newly developed high-risk human papillomavirus (hrHPV) ReadyMix qPCR Kit for detecting hrHPV in urine samples while genotyping for HPV-16, HPV-18, and HPV-52. A total of 876 samples were used to assess the performance of the hrHPV ReadyMix qPCR Kit in detecting hrHPV in standard cervical swab samples compared with that of the Roche Cobas 6800 HPV system. hrHPV detection in urine was compared with that in corresponding paired cervical swab samples. The sensitivity of the hrHPV ReadyMix qPCR Kit for HPV detection in cervical swab samples was 96.55%, and the specificity was 99.87%. Despite higher cycle threshold (Ct) values, urine samples demonstrated 80.88% sensitivity and 100.00% specificity compared with cervical swab samples. Our method enables population-based hrHPV analysis, with a 6.62% HPV prevalence determined via cervical swab samples and a 6.28% HPV prevalence determined via urine samples. Furthermore, the hrHPV ReadyMix qPCR Kit presented comparable HPV type distributions in urine samples and the ability to genotype HPV-16 and HPV-18 to those obtained via the Roche Cobas 6800 HPV system. Self-collected urine samples tested using the hrHPV ReadyMix qPCR Kit demonstrated a diagnostic accuracy of 98.48% for detecting hrHPV. This study highlights the potential of the hrHPV ReadyMix qPCR Kit to enhance cervical cancer screening, offering valuable insights for future interventions.

**IMPORTANCE:**

This study demonstrated that urinary detection of high-risk HPV DNA using the hrHPV ReadyMix qPCR Kit is an effective alternative for hrHPV screening. This non-invasive approach holds significant potential for large-scale screening, particularly in underserved populations. Integrating urine-based hrHPV testing into screening programs could improve early detection efforts, thereby enhancing the prevention and control of cervical cancer in low-resource settings.

## INTRODUCTION

Cervical cancer is the fourth most prevalent type of cancer among women, with approximately 604,127 cases and 341,831 fatalities worldwide in 2020 (1). The distribution of cervical cancer cases varies globally, with developing countries accounting for 85% of deaths (2). In Indonesia, approximately 36,633 cases and 21,003 deaths related to cervical cancer occur annually (3). However, the disease is preventable and treatable if it is detected early. Given that more than 99% of precancerous lesions (cervical dysplasia) and cervical carcinomas are caused by high-risk HPV (hrHPV) (4), tests for detecting hrHPV, as the etiological factor of cervical cancer, have emerged as a primary early screening method because of their greater sensitivity than the Pap test for detecting precancerous cervical lesions (5, 6). It also offers the advantages of extended screening intervals from once every 3 years to once every 5 years, as well as eliminating the need for skilled cytopathologists to interpret the results (4, 5).

However, the implementation of HPV-based diagnostics in emerging countries involves many challenges, such as high costs of screening tests, shortages of appropriate diagnostic kits, and screening participation rates lower than 10% (7, 8). To increase the low participation rates, a self-sampling approach combining the detection of HPV in urine samples with the utilization of the currently available qPCR instruments was developed as an alternative to the cervical swab-based HPV screening method. The use of urine samples offers several advantages, such as increased privacy during the sample collection process, reduced discomfort and pain, and ease of use. Furthermore, previous studies have shown that urine samples have promising accuracy for HPV detection (9–11). To address these needs, the hrHPV ReadyMix qPCR (ReadyMix) Kit was developed to detect 14 hrHPV types and can genotype HPV-52, HPV-16, and HPV-18 in both cervical swab samples and urine samples. HPV-16 and HPV-18 account for approximately 70% of all cervical cancer cases. In addition, HPV-52 has the highest prevalence in Southeast Asian countries (4, 12). Our study aimed to evaluate the performance of the ReadyMix Kit in detecting hrHPV in urine samples compared with standard cervical swab samples and to compare its performance with that of the Roche Cobas 6800 HPV system. The insights gained from this study will contribute to efforts in achieving effective screening measures, not only in Indonesia but also in other countries with similar settings, thus reducing the cervical cancer burden.

## MATERIALS AND METHODS

### Study design and population

This cross-sectional diagnostic test accuracy study was conducted based on HPV detection results from sexually active women aged 20–50 years residing in three major Indonesian cities: Jakarta, Bandung, and Semarang. The minimum number of participants was determined based on an anticipated diagnostic sensitivity of 90% and an hrHPV prevalence of 4.4% (13), yielding a requirement for a minimum of 786 participants (14). The study focused on women who sought healthcare services for general medical check-ups, Pap smears, or Visual Inspection with Acetic Acid (VIA) tests and agreed to participate in the study. The subjects were asked to provide urine and cervical swab samples. Pregnant women, women who were currently menstruating, and women who had already received the HPV vaccine were excluded from the study. Participants were recruited from July to October 2022.

### Specimen collection

The participants were first instructed to collect their first-void urine, which was collected in a urine container at any time of the day. The cervical swabs were then taken with a physician’s assistance via a cervical brush and preserved in PreservCyt solution (Hologic, Belgium). Both samples were kept at a temperature between 2°C and 8°C and sent to the Clinical Pathology Department laboratory at the Cipto Mangunkuso National Hospital for further analysis.

### DNA extraction

DNA was extracted from both the urine and cervical swab samples via a Zybio nucleic acid extraction kit (Zybio Inc., China) according to the manufacturer’s instructions. The samples were simultaneously run in the Cobas 6800 HPV system (Roche, Switzerland), where extraction and amplification were performed automatically. The integrity of the extracted DNA was assessed by detecting the β-globin gene later in the qPCR stage.

### HPV qPCR and genotyping protocol

The purified DNA was subjected to qPCR assays via ReadyMix performed in the CFX96 System (Bio-Rad Lab, CA, USA) and via the Cobas 6800 HPV system (Roche, Switzerland) (Cobas), performed directly in the system. ReadyMix was developed by Nusantics and commercialized as the PathoScan hrHPV qPCR Kit (Nusantics, Indonesia) and CerviScan HPV-hr qPCR Kit (PT Biofarma, Indonesia). The hrHPV ReadyMix qPCR Kit targets the E6‒E7 region to identify 14 hrHPV types while concurrently differentiating among HPV-16, HPV-18, and HPV-52. Other HPV types targeted include HPVs 31, 33, 35, 39, 45, 51, 56, 58, 59, 66, and 68, which are collectively detected in one fluorescence channel. Both tests detect the human β-globin gene as an internal control (IC) to monitor the success of the extraction and qPCR procedure. The Cobas assay was performed according to the manufacturer’s instructions. The ReadyMix reagent was premixed in a single tube and thus could be directly aliquoted into the qPCR well at a volume of 15 µL for a 20 µL qPCR. This kit was then used as per the manufacturer’s recommendation, with PCR thermal cycles consisting of 95°C initial denaturation for 2 minutes and 45 cycles of 1 second denaturation at 95°C and 10 seconds of annealing/extension at 60°C. The cutoff cycle threshold (Ct) value for a sample to be considered positive is 40.

In samples that showed discordant results between the two testing methods or between different sample types, the presence of HPV DNA was confirmed through amplicon sequencing via next-generation sequencing (NGS) technology. The DNA libraries were amplified via PCR, prepared via a Nextera XT index kit v2 (Illumina, CA, USA), and purified via AmPure XP beads (Beckman-Coulter, CA, USA). Paired-end (2 × 150 bp) sequencing was performed via the Illumina MiSeq Micro v2 reagent kit (Illumina, CA, USA) on the MiSeq platform. The sequencing primers used have been previously validated through alignment between the sequencing results of synthetic genes and their corresponding references. The reads were demultiplexed based on their index, resulting in a pair of forward and reverse fastq files for each sample. The downstream data analysis was performed via the QIIME2 platform (15). The primer sequences were trimmed, and the reads were filtered based on their length via Cutadapt (16), with a cutoff length of 20. Forward and reverse reads were merged via vsearch (17), allowing a maximum of 5 mismatches. In this step, a minimum merged read length of 30 for E6–E7 was enforced. Merged reads were quality-filtered based on their Q score with a cutoff value of 30 prior to dereplication. The reads were clustered *de novo* to produce operational taxonomic units with 99% similarity. Chimeric sequences were filtered out via the vsearch uchime-denovo tool. The resulting sequences were classified into HPV types via mapping to HPV reference sequences obtained from Papillomavirus Episteme (PaVE) (https://pave.niaid.nih.gov/). Mapping was performed via BLASTn with a minimum identity of 90% and a minimum word size of 20 (18).

### Statistical analysis

The data were assessed for normality via the Shapiro‒Wilk test. Categorical variables are presented as percentages. For measures of central tendency, the mean ± standard deviation (SD) was used for normally distributed data, whereas the median (interquartile range; IQR) was used for nonnormally distributed data. The sensitivity, specificity, positive predictive value (PPV), negative predictive value (NPV), and diagnostic accuracy were calculated to evaluate the diagnostic performance. Cohen’s kappa coefficient (κ) was used to determine concordance between compared tests or samples (19). Landis and Koch classified kappa values as follows: <0.00 as “poor,” 0.00–0.20 as “slight,” 0.21–0.40 as “fair,” 0.41–0.60 as “moderate,” 0.61–0.80 as “substantial,” and 0.81–1.00 as “almost perfect” (20). The Wilcoxon matched-pairs signed rank test was performed to compare the qPCR Ct values between the urine and cervical swab samples. All the statistical analyses were executed via GraphPad statistical software version 10.0.2 (GraphPad, San Diego, CA, USA).

## RESULTS

### Summary of subject participation and data validity

**Fig 1:**
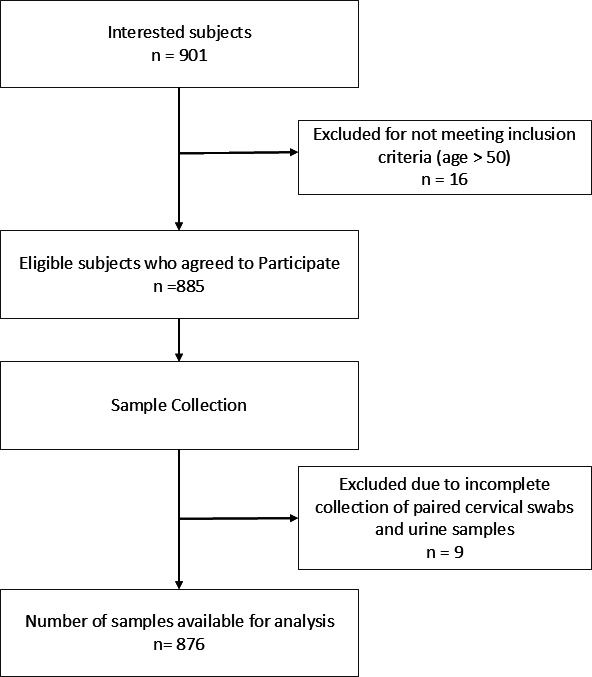
Flow chart describing the study subjects’ participation.

Between July and October 2022, 901 women who visited several healthcare facilities in Jakarta, Bandung, and Semarang for general medical check-ups, Pap smears, or visual inspection with acetic acid (VIA) tests and expressed interest in participating in this study were evaluated for eligibility. Sixteen individuals aged older than 50 years were subsequently excluded. Among the remaining 885 participants who provided samples, nine were ineligible because of incomplete collection of paired cervical swab and urine samples. Therefore, 876 subjects were included in this study (Fig. 1), consisting of 157 (17.92%), 346 (39.50%), and 373 (42.58%) subjects in the 20–29, 30–39, and 40–50 year age groups. The mean age of the subjects was 37.23 ± 7.28 years. A total of 385 (43.95%), 355 (40.53%), and 136 (15.53%) subjects were from Jakarta, Bandung, and Semarang, respectively.

Among the 876 cervical swab and urine samples tested with the Cobas system, 24 cervical swab (2.74%) and 206 urine samples (23.52%) yielded invalid results (Table 1). Conversely, ReadyMix produced fewer invalid results, with only 2 cervical swab (0.23%) and 19 urine samples (2.17%) yielding invalid results (Table 1).

**TABLE 1:** Summary of subject participation and data validity

Results	ReadyMix		Cobas	
	Cervical swab	Urine	Cervical swab	Urine
Total subjects	876	876	876	876
Subjects with complete test result	874	857	852	670
Invalid results	2	19	24	206
Invalid rate	0.23%	2.17%	2.74%	23.52%

### The hrHPV ReadyMix qPCR Kit showed a strong agreement with the Cobas 6800 HPV system in high-risk HPV detection

In this study, HPV detection specifically refers to hrHPV detection. The analytical performance of ReadyMix was previously characterized based on a linearity assay and the limit of detection (LoD), which yielded satisfactory results (Supplementary Materials: Fig. S1 and S2). We first sought to determine the diagnostic performance of ReadyMix in HPV detection via the use of cervical swab samples as a standard method. The accuracy was 98.94% (95% CI: 98.00%–99.44%), with 96.15% sensitivity (95% CI: 87.02%–99.32%) and 99.13% specificity (95% CI: 98.2–99.58) (Table 2).

**TABLE 2:** HPV detection in cervical swab samples via ReadyMix versus the Cobas system

Cervical swab (ReadyMix)	Cervical swab (Cobas)			k-coefficient
	Positive	Negative	Total	
Positive	50	7	57	0.91
Negative	2	793	795	
Total	52	800	852	

Effect size	Value (%)	95% CI (%)
Sensitivity	96.15	87.02–99.32
Specificity	99.13	98.2–99.58
Positive predictive value	87.72	76.75–93.92
Negative predictive value	99.75	99.09–99.96
Accuracy	98.94	98.00–99.44

For the samples with discordant results, we found that six out of the seven false positives were confirmed to be true positives via the NGS results (Supplementary Materials: Table S1). To obtain a clearer report of the performance of this assay, we then incorporated NGS data, which resulted in 96.55% (95% CI: 88.27%–99.39%) sensitivity and 99.87% (95% CI: 99.29%–99.99%) specificity (Table 3). The Cohen’s kappa coefficient for agreement between methods was 0.97.

**TABLE 3:** HPV detection in cervical swab samples via ReadyMix versus Cobas and NGS

Cervical swab (ReadyMix)	Cervical swab (Cobas & NGS)			k-coefficient
	Positive	Negative	Total	
Positive	56	1	57	0.97
Negative	2	793	795	
Total	58	794	852	

Effect size	Value (%)	95% CI (%)
Sensitivity	96.55	88.27–99.39
Specificity	99.87	99.29–99.99
Positive predictive value	98.25	90.71–99.91
Negative predictive value	99.75	99.09–99.96
Accuracy	99.65	98.97–99.90

### HPV detection results in urine samples were closely similar to those in cervical swab samples

To validate the use of urine samples for HPV detection, we compared their performance with that of a well-established cervical swab test. The NGS-corrected data were used to analyze the performance of ReadyMix. The NGS results are included in the supplementary data (Supplementary Materials: Table S1; Fig. S3). The Cohen’s kappa coefficient for agreement between cervical swab and urine samples was 0.89, with a sensitivity of 80.88% (95% CI: 69.99–88.47%) and a specificity of 100% (95% CI: 99.51%–100%) (Table 4). This result demonstrated enhanced accuracy of ReadyMix in detecting HPV in urine compared with that of the Cobas system (Supplementary Materials: Table S2).

**TABLE 4:** HPV detection via ReadyMix in urine samples versus cervical swab samples and NGS

Urine (ReadyMix)	Cervical swab (ReadyMix & NGS)			k-coefficient
	Positive	Negative	Total	
Positive	55	0	55	0.89
Negative	13	787	800	
Total	68	787	855	

Effect size	Value (%)	95% CI (%)
Sensitivity	80.88	69.99–88.47
Specificity	100.00	99.51–100
Positive predictive value	100.00	93.47–100
Negative predictive value	98.38	97.24–99.05
Accuracy	98.48	97.42–99.11

### Urine samples retained sensitivity across cervical swab samples with low, moderate, and high Ct values

**Fig 2:**
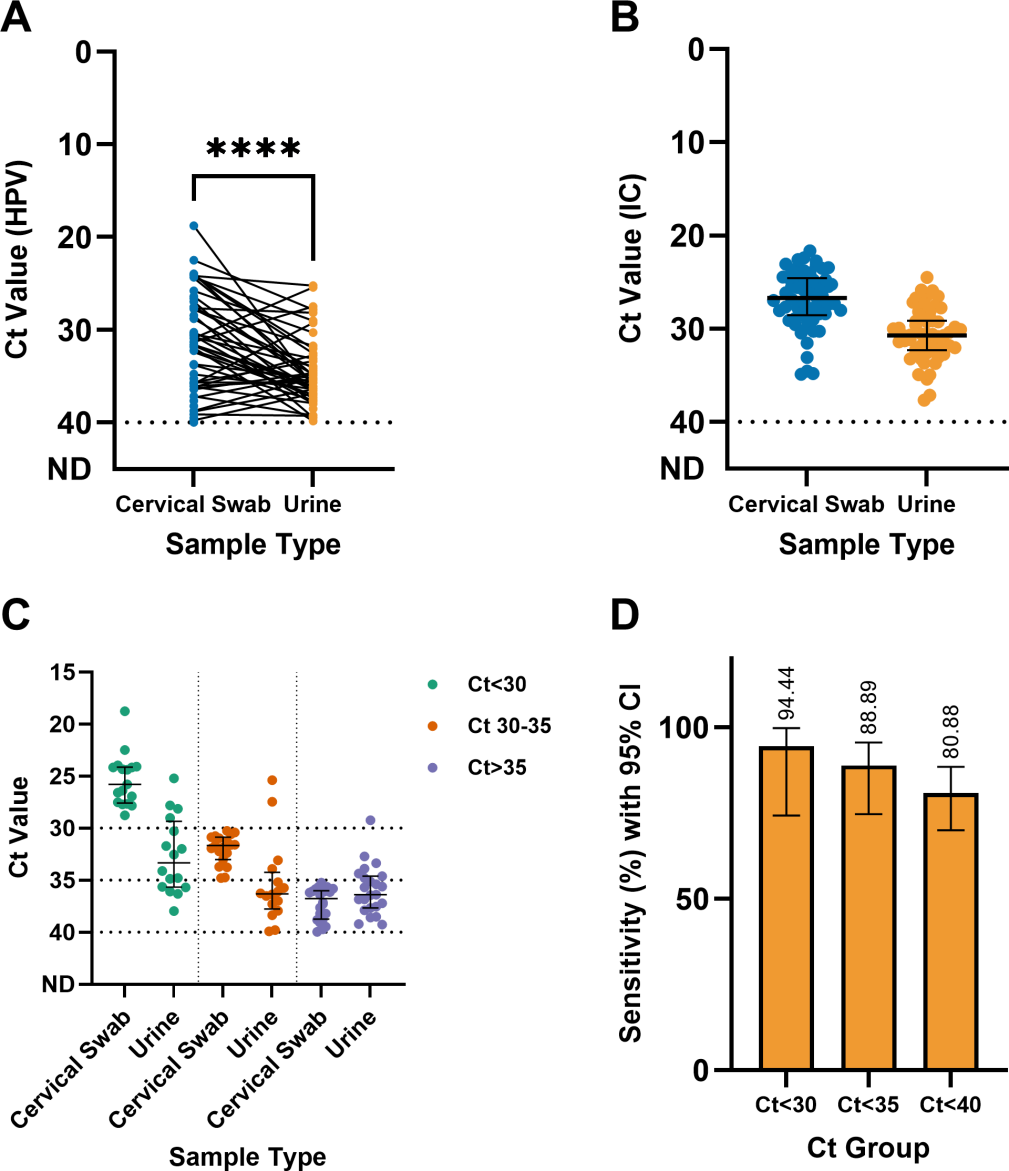
The Ct values of HPV detection in cervical swab and urine samples were significantly different (Wilcoxon matched-pairs signed rank test, *P* value: <0.0001), with cervical swab samples tending to have a lower Ct value (**A**). This finding was supported by a similar pattern of Ct values for IC detection (**B**). However, the Ct value differences between cervical swab and urine samples decreased as the sample’s Ct value increased (**C**), and the sensitivity of HPV detection in urine samples was maintained at >80% across different viral load groups (**D**).

When the HPV Ct values of paired cervical swab and urine samples were analyzed, a significant difference was detected between the two samples based on the Wilcoxon matched-pairs signed rank test (*P* value < 0.0001) (Fig. 2A). The cervical swab samples presented a lower median HPV Ct value (31.89 [8.41]) than did the urine samples (35.69 [3.85]), resulting in a median difference of 3.80. The Ct value of IC detection also showed a similar pattern, whereby cervical swab samples had a lower median (26.685 [3.82]) than did urine samples (30.69 [3.18]) (Fig. 2B).

The median differences in the HPV Ct values of the two sample types followed a pattern where the difference tended to decrease as the Ct values increased. For cervical swab samples, the low Ct group (Ct <30) had a median Ct value of 26.08 (3.51), whereas the corresponding urine samples had a median Ct value of 34.11 (6.24) (Fig. 2C). For the cervical swab samples, the medium Ct group (Ct 30–35) had a median Ct value of 31.63 (1.53), whereas the corresponding urine samples had a median Ct value of 36.26 (3.36) (Fig. 2C). For the cervical swab samples, the high Ct group (Ct >35) had a median Ct value of 36.76 (2.66), whereas the paired urine samples had a median Ct value of 36.39 (3.04) (Fig. 2C). Thus, the median differences between urine and cervical swab samples for the low-, medium-, and high-Ct groups were 8.03, 4.63, and 0.37, respectively. Despite the wider median gap at a lower Ct value and the tendency to produce higher Ct values, the sensitivity of urine samples was maintained above 80% across all Ct groups, ranging from 80.88% when considering all samples to 99.44% when considering only samples with Cts < 30 (Fig. 2D).

### The hrHPV ReadyMix qPCR Kit and Cobas 6800 HPV system produced comparable HPV prevalence patterns across different age groups and locations

**Fig 3:**
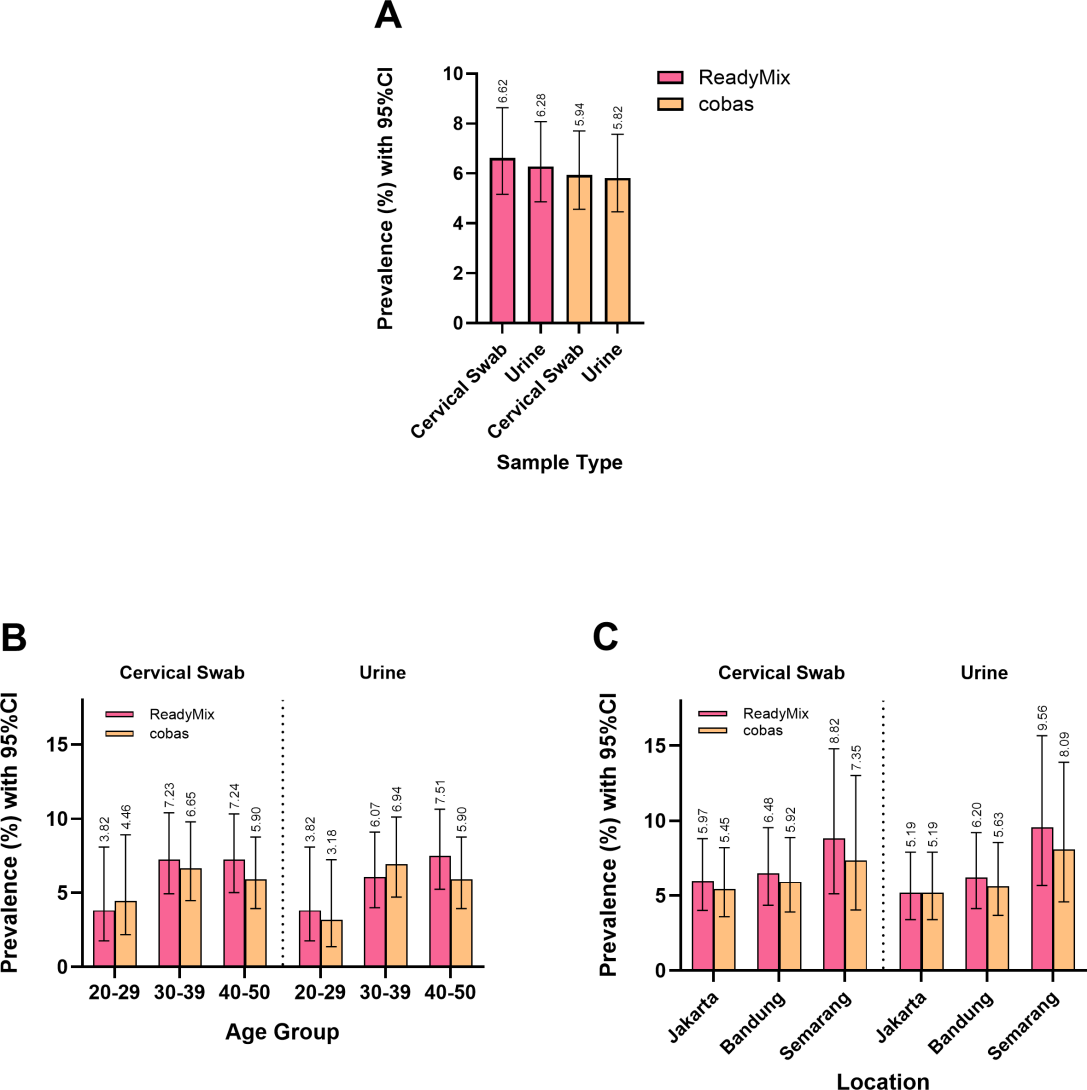
ReadyMix identified a higher HPV prevalence, as confirmed by NGS (**A**). The prevalence distribution across age groups revealed that ReadyMix had a greater prevalence in the 40- to 50-year age group, whereas the Cobas system revealed a greater prevalence in the 30- to 39-year age group (**B**). Both tests identified the 20- to 29-year age group as having the lowest prevalence. The prevalence distribution across locations showed a consistent pattern between ReadyMix and the Cobas system, with similar results observed for both the cervical swab and urine samples (**C**).

The prevalence of HPV in the study population was slightly greater when tested with ReadyMix than when tested with the Cobas system (Fig. 3A). ReadyMix revealed an HPV prevalence of 6.62% and 6.28% based on cervical swab and urine samples, respectively, whereas the Cobas system revealed an HPV prevalence of 5.94% and 5.82%, respectively. As previously described, most of the additional positive samples were shown to be true positives by NGS, suggesting the superior performance of ReadyMix. However, we found a similar HPV prevalence according to the two tests across different age groups and different locations.

The 20- to 29-year age group had the lowest HPV prevalence according to both tests based on cervical swab (ReadyMix: 3.82%; Cobas: 4.46%) and urine (ReadyMix: 3.82%; Cobas: 3.18%) samples (Fig. 3B). ReadyMix had a marginally greater prevalence in the 40- to 50-year age group (cervical swab: 7.24%, urine: 7.51%) than in the 30- to 39-year age group (cervical swab: 7.23%, urine: 6.07%). Conversely, the Cobas system revealed a higher prevalence in the 30- to 39-year group (cervical swab: 6.65%, urine: 6.94%) than in the 40- to 50-year group (cervical swab: 5.90%, urine: 5.90%) (Fig. 3B). This contrasting finding was mostly the result of several samples (five cervical swab and five urine samples) from the 40- to 50-year group that presented negative according to the Cobas system but were shown to be positive by ReadyMix and confirmed by NGS (Supplementary Materials: Table S1).

A comparison of the prevalence across different locations revealed that Semarang had the highest HPV prevalence based on cervical swab samples (ReadyMix: 8.89%; Cobas: 7.35%), followed by Bandung (ReadyMix: 6.48%; Cobas: 5.92%), whereas Jakarta presented the lowest HPV prevalence (ReadyMix: 5.97%; Cobas: 5.45%) (Fig. 3C). This similar pattern was also observed in the urine samples, with an HPV prevalence of 9.56% and 8.09% in Semarang according to ReadyMix and the Cobas system, respectively, followed by Bandung (ReadyMix: 6.20%; Cobas: 5.63%), whereas Jakarta continued to have the lowest prevalence among the three locations (ReadyMix: 5.19%; Cobas: 5.19%).

### A similar HPV prevalence was observed for both urine samples and cervical swabs

**Fig 4:**
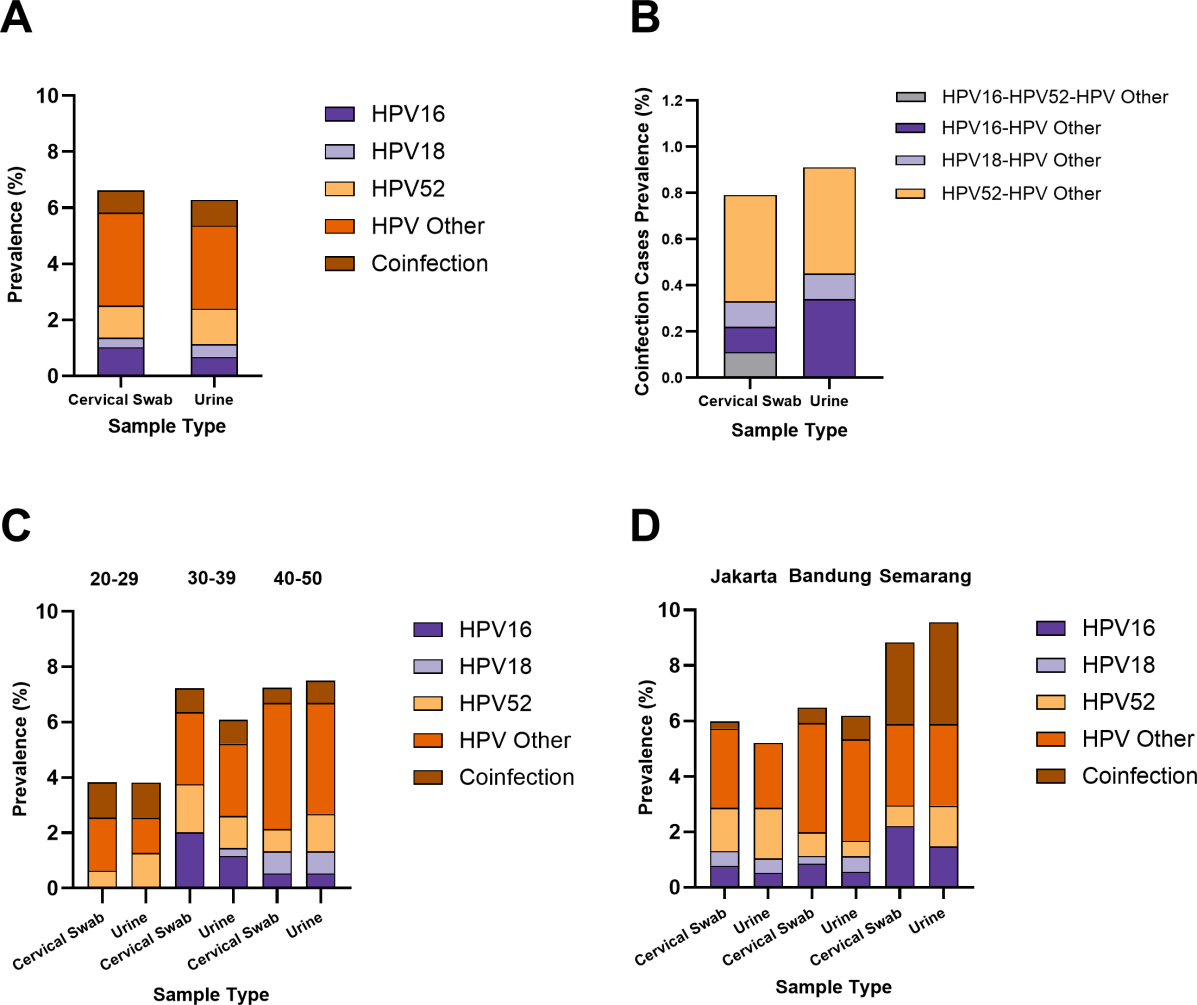
The proportions of each HPV type/group found in the urine samples were comparable to those in the cervical swab samples (**A**). Urine samples also enabled coinfection case detection, showing patterns consistent with those observed in cervical swab samples, except for the absence of one case of positive triple-types/group (**B**). The HPV type/group proportions in urine samples were comparable to those in cervical swab samples across different age groups, with one HPV-18 case in the 30- to 39-year age group detected in urine and confirmed by NGS. HPV-16 and HPV-18 were not detected in the 20- to 29-year age group across either sample type (**C**). A location-based analysis revealed consistent HPV proportions between the urine and cervical swab samples, except for the absence of one coinfection case in the urine samples from Jakarta (**D**).

In addition to the similar HPV prevalence, the pattern of the HPV type/group proportion in the urine samples was also comparable to that in the cervical swab samples, with HPV-others having the highest proportion (cervical swab: 50.00%; urine: 47.27%), followed by HPV-52 (cervical swab: 17.24%; urine: 20.00%), HPV-16 (cervical swab: 15.52%; urine: 10.91%), and HPV-18 (cervical swab: 5.17%; urine: 7.27%) (Fig. 4A). There were also some co-infection cases, accounting for 12.07% and 14.55% of the cases of HPV detected in the cervical swab and urine samples, respectively. Most coinfections involved HPV-52 and HPV-other combinations (cervical swab: 57.14%; urine: 50.00%) (Fig. 4B). The proportions of HPV-16–HPV-other, HPV-18–HPV-other, and HPV-16–HPV-52–HPV-other in the cervical swab samples were 14.28%. In the urine samples, however, the proportion of HPV-16-HPV-other coinfections (37.50%) was greater than that of HPV-18–HPV-other coinfections (12.50%). In the cervical swab samples, the sample with triple-positive signals (HPV-16–HPV-52–HPV-other) was identified as HPV-16–HPV-other in the corresponding urine sample. A subset of coinfection samples was further confirmed to contain the specific HPV types detected (HPV-16/18/52) via NGS, as well as those identified as HPV-other, including HPV-31, HPV-33, HPV-51, HPV-56, and HPV-58 (Supplementary Materials: Table S1; Fig. S3).

The similarity in HPV proportion patterns between urine and cervical swab samples was consistently observed across different age groups (Fig. 4C). According to the cervical swab samples, HPV-other was the main HPV type detected (20- to 29-year age group: 50.00%, 30- to 39-year age group: 36.00%, and 40- to 50-year age group: 62.96%). The urine samples also showed a similar pattern (30- to 39-year age group: 42.86%; 40- to 50-year age group: 53.57%), except for the 20- to 29-year age group, where the proportion of HPV-other was the same as that of HPV-52, and that of coinfection cases was 33.33%. In the 20- to 29-year age group, neither HPV-16 nor HPV-18 was detected. In the 30- to 39-year age group, the proportions of HPV-52 and HPV-16 were similar in the cervical swab samples (24.00% and 28.00%, respectively) and urine samples (19.05%). A small proportion of HPV-18 was detected in the urine samples of this group (4.76%), whereas no HPV-18 was detected in the cervical swab samples. The presence of HPV genetic materials was confirmed with NGS, which revealed that the detection of HPV-18 was not a false-positive result. In the 40- to 50-year age group, cervical swab and urine samples presented similar proportions of HPV-16 (cervical swab: 7.41%; urine: 7.14%) and HPV-18 (cervical swab: 11.11%; urine: 10.71%), whereas the proportion of HPV-52 was greater in the urine samples (17.86%) than in the cervical swab samples (11.11%).

In the analysis of HPV prevalence by location, HPV-other remained dominant, except in Semarang, where the proportion was the same as that of coinfection cases (33.33%), as determined in cervical swab samples (Fig. 4D). Semarang, in fact, had the highest proportion of coinfection cases determined in urine samples (38.46%) when compared across all locations (Jakarta: 0.00%; Bandung: 13.64%). HPV-52 accounted for the second largest proportion of cases in Jakarta; in both cervical swab (26.09%) and urine samples (35.00%), while in Semarang, the proportion was the same as that of HPV-16 (at 15.38%) in urine samples or lower in cervical swab samples (HPV-52: 8.33%; HPV-16: 25.00%). HPV-18 was detected in both cervical swab and urine samples from Jakarta (cervical swabs: 8.7%; urine: 10%) and Bandung (cervical swabs: 4.35%; urine: 9.09%), while no cases were detected in Semarang. A single case of coinfection was identified in Jakarta through a cervical swab sample (4.35%), with no corresponding detection in the urine sample. By contrast, Bandung revealed a greater proportion of coinfections in urine samples than in cervical swab samples, with one additional case identified (cervical swabs: 8.70%; urine: 13.64%).

### HPV-16 and HPV-18 genotyping results obtained with the hrHPV ReadyMix qPCR Kit were similar to those obtained with the Cobas 6800 HPV system

**Fig 5:**
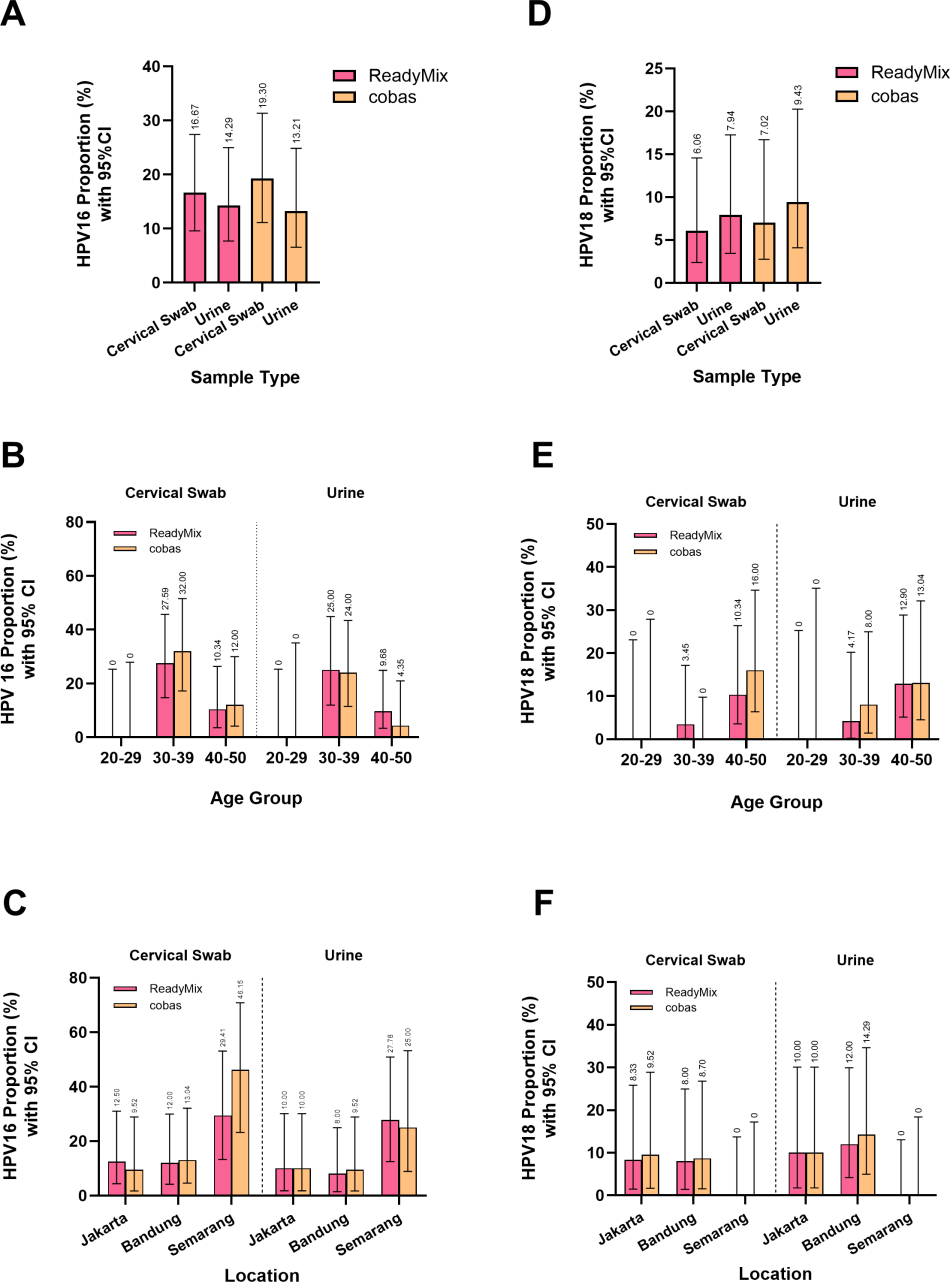
The proportions of HPV-16 detected by ReadyMix were comparable to those detected by the Cobas system, with higher proportions observed in cervical swab samples than in urine samples (**A**). This similarity was also observed across different age groups (**B**) and locations (**C**). HPV-16 was not detected in the 20 to 29-year age group across either sample type or kit. The overall proportion of HPV-18 was slightly greater in urine samples than in cervical swab samples for both ReadyMix and the Cobas system (**D**). However, in the 40- to 50-year age group, the Cobas system detected a slightly greater proportion of HPV-18 in the cervical swab samples than in the urine samples. HPV-18 was not detected in the 20- to 29-year age group across either sample type and kit (**E**). The HPV-18 proportions across different locations were consistent between ReadyMix and the Cobas system. HPV-18 was not detected in Semarang across either sample type and kit (**F**).

To further validate the performance of ReadyMix, we compared the genotyping ability of HPV-16 and HPV-18 with that of the Cobas system. The proportion of HPV-16-positive HPV samples was greater for the cervical swab (ReadyMix: 16.67%; Cobas: 19.30%) than for the urine samples (ReadyMix: 14.29%; Cobas: 13.21%) (Fig. 5A; Supplementary Materials: Table S4). The distribution of HPV-16 across the different age groups was consistent among all the samples tested by ReadyMix and the Cobas system, with the highest prevalence observed in the 30- to 39-year age group (cervical swab: ReadyMix: 27.59%, Cobas: 32.00%; urine: ReadyMix: 25.00%, Cobas: 24.00%), followed by the 40- to 50-year age group (cervical swab: ReadyMix: 10.34%, Cobas: 12.00%; urine: ReadyMix: 9.68%, Cobas: 4.35%), whereas no HPV-16 was detected in the 20- to 29-year age group (Fig. 5B; Supplementary Materials: Table S4).

In terms of HPV-16 prevalence from the perspective of location, Cobas detected a greater proportion of HPV-16 (46.15%) in cervical swab samples in Semarang than did ReadyMix (29.41%) (Fig. 5C). The total number of HPV-positive samples detected with the Cobas system was smaller than that detected with ReadyMix (ReadyMix: 17 HPV-positive samples; Cobas: 13 HPV-positive samples), whereas the number of HPV-16-positive samples detected with both ReadyMix and the Cobas system was similar (ReadyMix: 5 HPV-16-positives; Cobas: 6 HPV-16-positives), resulting in a difference in HPV-16 prevalence. On the other hand, the urine samples presented more consistent results, with proportions of 27.78% and 25.00% for HPV-16 cases when tested with ReadyMix and Cobas, respectively. The cervical swab samples tested with ReadyMix had similar proportions of HPV-16 cases in Jakarta (12.50%) and Bandung (12.00%), whereas a slightly lower proportion of HPV-16 cases in Jakarta was obtained when the samples were tested with Cobas (Jakarta: 9.52%; Bandung: 13.04%). On the other hand, Bandung had a slightly lower HPV-16 proportion than did Jakarta when the urine samples were tested with ReadyMix (Jakarta: 10.00%; Bandung: 8.00%) and Cobas (Jakarta: 10.00%; Bandung: 9.52%).

Both tests revealed a greater prevalence of HPV-18 in urine samples (ReadyMix: 7.94%; Cobas: 9.43%) than in cervical swab samples (ReadyMix: 6.06%; Cobas: 7.02%) (Fig. 5D). The proportion of HPV-18-positive samples, in general, increased with increasing age (Fig. 5E). The 40- to 50-year age group had the highest proportion of HPV-18 cases (ReadyMix: 10.34% and Cobas: 16.00%) in the cervical swab samples, with proportions of 12.90% (ReadyMix) and 13.04% (Cobas) in the urine samples. There were no samples in which HPV-18 was found in the 20-29-year age group.

The location-based HPV proportion analysis indicated that the HPV-18 proportion was slightly greater in Jakarta (ReadyMix: 8.33%; Cobas: 9.52%) than in Bandung (ReadyMix: 8.00%; Cobas: 8.70%) based on the cervical swab samples (Fig. 5F). On the other hand, urine samples presented a slightly greater proportion of HPV-18 in Bandung (ReadyMix: 12.00%; Cobas: 14.29%) than in Jakarta (ReadyMix: 10%; Cobas: 10%). There were no samples in Semarang that were positive for HPV-18.

### Cervical swab and urine samples demonstrated a similar pattern in terms of the proportion of HPV-52 cases

**Fig 6:**
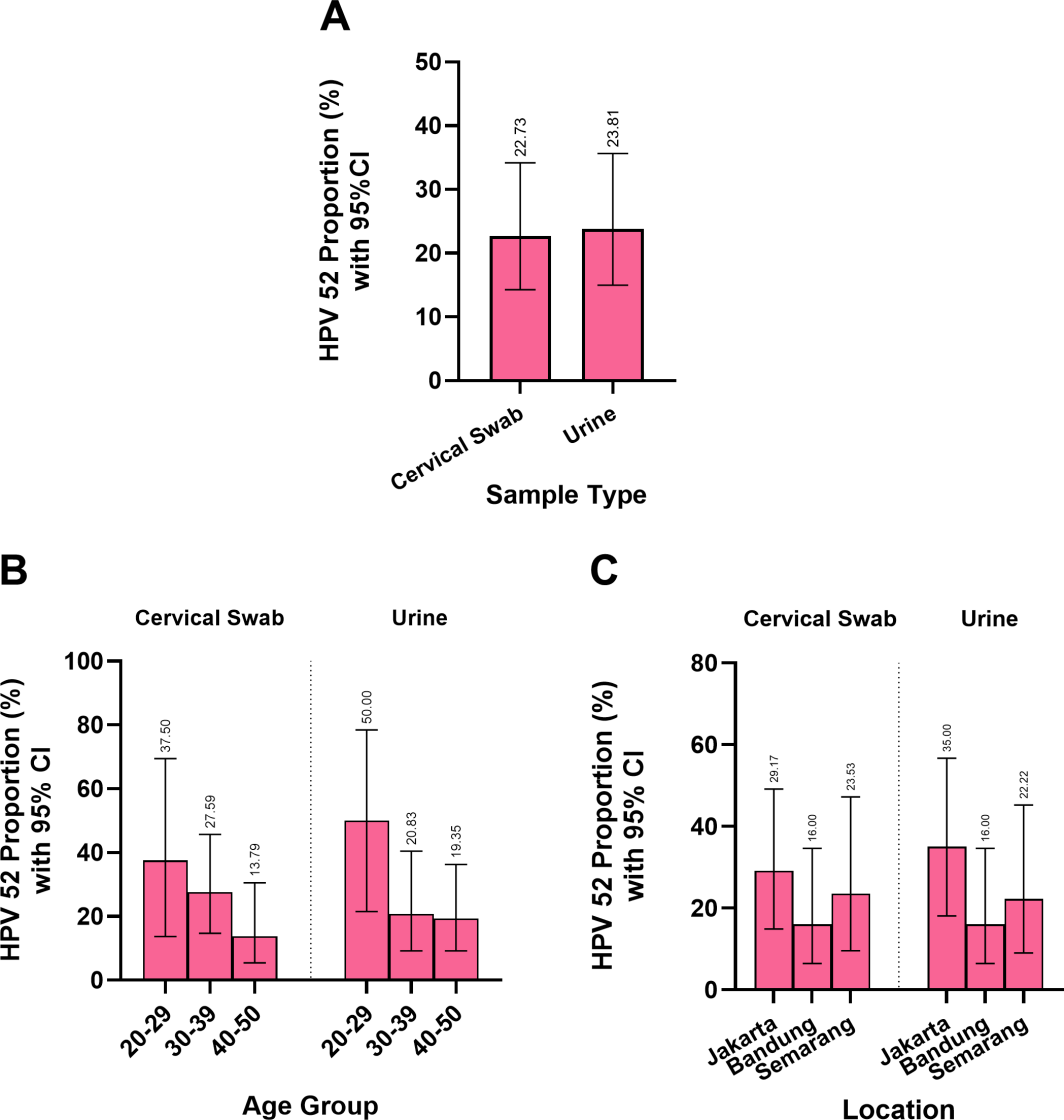
The overall proportions of HPV-52 detected in the cervical swab and urine samples were similar (**A**). Both sample types presented the highest proportion of HPV-52 in the 20- to 29-year age group, and the proportion decreased with increasing age (**B**). Both sample types identified Jakarta as having the highest HPV-52 proportion, followed by Semarang and Bandung (**C**).

Owing to its ability to genotype HPV-52, the ReadyMix assay identified comparable proportions of HPV-52 in cervical swab and urine samples (cervical swabs: 22.73%, urine: 23.81%) (Fig. 6A). Age-stratified analysis revealed that both sample types had the highest proportion of HPV-52-positive individuals in the 20- to 29-year group (cervical swab: 37.50%, urine: 50.00%), with a tendency to decrease in the older age groups: 30–39 years (cervical swab: 27.59%, urine: 20.83%) and 40–50 years (cervical swab: 13.79%, urine: 19.35%) (Fig. 6B).

A location-based analysis also revealed consistent results across both sample types, with the highest proportion of HPV-52 cases in Jakarta (cervical swab: 29.17%, urine: 35.00%), followed by Semarang (cervical swab: 23.53%, urine: 22.22%) and Bandung (cervical swab: 16.00%, urine: 16.00%) (Fig. 6C).

## DISCUSSION

Our study demonstrated the good performance of the ReadyMix Kit in detecting HPV via both standard cervical swab and urine samples, with diagnostic accuracies of 99.65% and 98.48%, respectively. These results were also validated by NGS, in which the samples with discordant results were sequenced to confirm the presence of HPV genetic materials. We also confirmed that the prevalence of hrHPV in urine samples was similar to that in cervical swab samples, indicating the reliability of the urinary detection of hrHPV via the ReadyMix Kit in cervical cancer screening.

The convenience of using urine samples for HPV detection has garnered interest as an alternative to the conventional cervical swabbing method. Cervical examination is frequently seen as an uncomfortable and embarrassing procedure, particularly among women in developing countries, preventing them from being screened (21, 22). Nishimura et al. (23) reported that self-sampling is more acceptable, as it protects privacy and reduces anxiety (23). Furthermore, Zhao et al. (7) noted that women were more confident in correctly collecting urine samples than in collecting cervical samples (7). In addition, the use of self-collected urine samples reduces the need for skilled personnel and special supporting equipment for cervical swab sample collection.

Compared with conventional cervical swab samples, urine samples have been reported to show promising performance for HPV detection (9–11). Unlike its preference for the cervix and other anogenital areas, HPV has no inherent affinity for the urinary tract. Consequently, the detection of HPV in urine likely signifies secondary exfoliation originating from cervical or other anogenital lesions (24). Therefore, the use of first-void urine is crucial to acquire a greater quantity of exfoliated cells, which are likely to have a higher concentration of HPV DNA (9). In this study, we allowed the participants to collect first-void urine at any time of the day for convenience, as there is no disparity in HPV DNA concentrations between morning and subsequent samples as long as they are first-void samples (25).

A comprehensive analysis of multiple studies revealed that the combined sensitivity of hrHPV detection in urine samples using a range of nucleic acid tests was 77%, with a specificity of 88%, compared with that of cervical samples (9). Based on these findings, we observed higher sensitivity and specificity (80.88% and 100%, respectively) when ReadyMix was used. This enhanced performance may be attributed in part to the lower rate of invalid results for the urine samples. For context, in other assays, such as the Cobas assay, we observed a sensitivity of 77.05% and specificity of 99.66% for HPV detection in urine samples, along with higher invalid rates (Table 1; Supplementary Materials: Table S2). In comparison, similar studies by Torres-Rojas et al. utilized first-void urine samples collected from an indigenous population, reporting a concordance of 68.27% using the INNO-LiPA HPV Genotyping Extra II assay (Fujirebio) (26). Our study, however, demonstrated a higher concordance of 98.48% (Table 4). The ReadyMix assay was meticulously engineered for superior diagnostic performance, incorporating an advanced primer‒probe design, a high-fidelity DNA polymerase enzyme, and a custom‒optimized buffer—an extensive refinement specifically developed to maximize assay sensitivity and reliability (Supplementary Materials: Fig. S1 and S2). These enhancements underscore the advantages of ReadyMix in achieving high accuracy in HPV detection in urine samples, even within general population settings.

In addition to the satisfactory detection rates, the HPV genotypes detected in urine samples were comparable to those detected in cervical swab samples (Fig. 4). This finding aligns with the observations of Sahasrabuddhe et al., who reported a notably high concordance rate (97.1%) between HPV genotypes identified in urine samples and cervical lesion samples (24). This result highlights the potential of HPV detection in urine samples via ReadyMix as a reliable screening method.

The good performance of ReadyMix can also be shown by its similar HPV-16 and HPV-18 genotyping results to those of the Cobas system. This confirms the proficiency of ReadyMix in accurately determining HPV-16- and HPV-18-positive cases, providing clinicians with valuable information to guide subsequent measures. The 2019 American Society for Colposcopy and Cervical Pathology (ASCCP) risk-based management consensus guidelines suggest that additional evaluations, such as colposcopy, be performed in HPV-16 and HPV-18-positive patients, even when the cytology results are negative (27). HPV-52 genotyping, as an additional genotyping capability of ReadyMix, also helps in determining the HPV type with the highest proportion among the hrHPVs detected in Indonesia (28). Our study revealed a greater proportion of HPV-52 than of HPV-16 and HPV-18 in both cervical swab (HPV-52: 17.24%; HPV-16: 15.52%; HPV-18: 5.17%) and urine samples (HPV-52: 20.00%; HPV-16: 10.91%; HPV-18: 7.27%). These results are in line with those of a previous study in which HPV-52, HPV-16, and HPV-18 accounted for 23.2%, 18.0%, and 16.1%, respectively, of hrHPV-positive cases in Indonesia (28). Despite the 15-year gap, our findings continue to emphasize the necessity of a vaccine covering HPV-52, in addition to HPV-16 and HPV-18, for the Indonesian population.

The screening data from ReadyMix revealed an HPV prevalence of 6.28-6.62%, which is higher than that of 5.2% reported in a previous study conducted in Indonesia, where hrHPV accounted for 4.4% of HPV cases (13). Fortunately, this rate remains below the global prevalence of hrHPV, estimated at 10.4% (29). Unlike most studies where the HPV prevalence decreases with increasing age (30), we found a lower HPV prevalence in the 20- to 29-year age group (cervical swab: 3.82%; urine: 3.82%) than in the 30- to 39-year (cervical swab: 7.23%; urine: 6.07%) and 40- to 50-year age groups (cervical swab: 7.24%; urine: 7.51%). Moreover, in the 20- to 29-year age group, neither HPV-16 nor HPV-18 was detected, whereas HPV-52, which is more common in the general population, had the greatest proportion compared with the other age groups (Fig. 6B, Supplementary Materials: Table S4). Vet et al. (28) also reported that there was no decline in the prevalence of HPV in older subjects from Jakarta and Tasikmalaya, Indonesia (28). In addition, an increasing prevalence in older age groups in China was also reported by Yang et al. (31), with the highest overall HPV prevalence observed in the ≥50-year age group (10.53%), followed by the 30- to 49-year age group (8.29%), and the lowest prevalence in the <30-year age group (7.30%) (31). Several factors may account for this phenomenon, including a lower representation of younger individuals due to the randomized subject screening methodology used in this study. In addition, the reduced immunological function in older individuals may increase their susceptibility to HPV infection, compounded by prolonged exposure to risk factors over time (31). Based on these data, we suggest an increased effort in the diagnosis of HPV infection in older women, as they have a greater risk of cervical cancer progression and lower protection derived from vaccination (32).

We found that the prevalence of HPV was the highest in Semarang (cervical swab: 8.89%; urine: 9.56%), followed by Bandung (cervical swab: 6.48%; urine: 6.20%), and finally Jakarta (cervical swab: 5.97%; urine: 5.19%). Here, we can see that Jakarta, the largest metropolitan city, had the lowest HPV prevalence compared with Semarang, a metropolitan city with a larger rural area. Bandung, a metropolitan city positioned between the two cities (33), also presents a prevalence that falls between those of the two aforementioned cities. While Bandung and Jakarta presented relatively similar proportions of HPV-16 and HPV-18, Semarang presented a distinct pattern, with the highest proportion of HPV-16 and no HPV-18 detected (Fig. 5C and F). Notably, Jakarta had a higher proportion of HPV-52—a more common HPV type in the population—as observed consistently across both cervical swab and urine samples (Fig. 6C). Despite many lifestyle factors influencing the prevalence of HPV in communities (31), Sabeena et al. (34) reported that the infection rate in rural women is higher than that in urban women because of lower access to cancer screening and oncology care (34). This is a continuing problem for developing countries due to the inequality of access to healthcare services.

In our study, we identified multiple HPV-positive signals in 7 out of 58 HPV-positive cervical swab samples (12.07%) and 8 out of 55 HPV-positive urine samples (14.54%) (HPV-16/HPV-18/HPV-52/HPV-other). In addition, our NGS data (Supplementary Materials: Table S1) indicate that some samples positive for HPV-other may harbor multiple HPV types. HPV-positive individuals are often infected with multiple HPV types (35–37). However, the precise implications of this phenomenon for cervical cancer onset and progression remain elusive. While some studies suggest the lack of a clear correlation between multiple HPV infections and cervical histology (38, 39), others propose that multiple infections of specific types may synergistically increase the risk of cervical carcinoma (40, 41). However, some additional studies indicate that the disease risk is only similar to the sum of the estimated risk of individual types, with little to no evidence of synergistic interactions (36, 42). Considering that certain HPV types are inherently more carcinogenic than others (43–45), we suggest that prioritizing the detection of these more carcinogenic HPV types holds greater utility in initial screening protocols than attempting to differentiate among all HPV types within an infected individual.

Our study, which used urine samples for cervical cancer screening via the hrHPV ReadyMix Kit, has several strengths. In addition to including a high number of participants across multiple age groups, we also enrolled subjects from three major cities with varying metropolitan ratings and likely different lifestyles (32). This is particularly important to ensure that the method of HPV detection via urine samples applies to the Indonesian population. Our study design also ensured that the paired urine samples and cervical swabs were collected on the same day to reduce the variability of the results that might arise from different sampling times. In addition, urine samples were collected before the cervical swab samples to reduce bias from additional cell debris that might come from the swabbing procedure. We also implemented NGS to confirm the potential false-positive qPCR results to check for the presence of HPV in both cervical swab and urine samples, which resulted in the confirmation of HPV that was not detected by the Cobas system. However, the main weakness of our study is that we did not perform any visual inspection of the cervix to confirm disease status and its relevance to the qPCR results. These limitations should be overcome by future studies to obtain a better perspective of hrHPV conditions.

## Data Availability

The data sets supporting the conclusions of this article are included within the article and its supplementary materials. Additionally, we provide access to raw data collected during a clinical trial assessing the effectiveness of urinary detection of high-risk HPV DNA to improve cervical cancer screening in developing countries at https://doi.org/10.6084/m9.figshare.26362630.

## SUPPLEMENTAL MATERIAL

Supplemental material spectrum.01938-24-s0001.docx Figures S1 to S3; Tables S1 to S4.

ASM does not own the copyrights to Supplemental Material that may be linked to, or accessed through, an article. The authors have granted ASM a non-exclusive, world-wide license to publish the Supplemental Material files. Please contact the corresponding author directly for reuse.
