# Supplementary material for: Urinary detection of high-risk HPV DNA to enhance cervical cancer screening in developing countries
Source: Microbiol Spectr. 2025 Jul 18;13(9):e01938-24. doi: 10.1128/spectrum.01938-24 (PMC12403565; doi:10.1128/spectrum.01938-24)
Supplement: Supplemental material — Figures S1 to S3; Tables S1 to S4. [file spectrum.01938-24-s0001.docx]

**Supplementary Materials**

Of

**Urinary Detection of High-Risk HPV DNA to Enhance Cervical Cancer Screening in Developing Countries**

Novia Syari Intan, Revata Utama, Dewi Wulandari, Reiva Wisdharilla, Shafira Mutia Khanza, Muhamad Rifki Ramadhan, Indah Suci Widyahening, Neni Nurainy, Rini Mulia Sari, Andrijono

| A) | 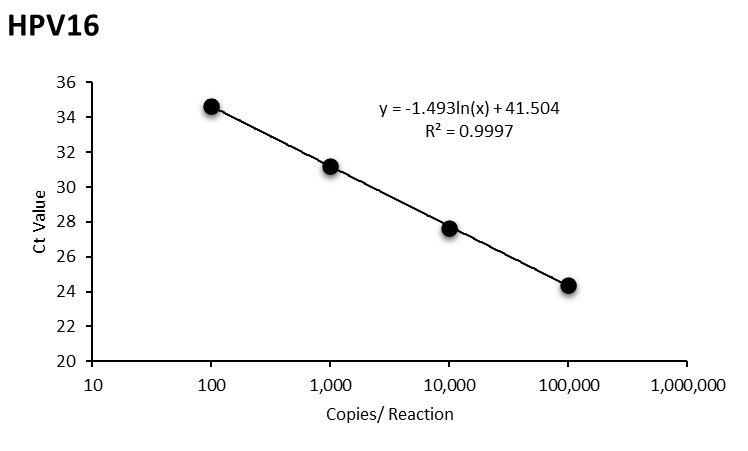 | H) | 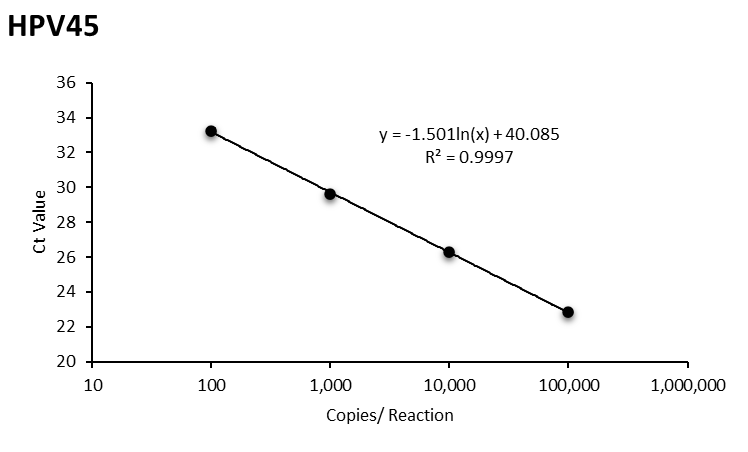 |
| --- | --- | --- | --- |
| B) | 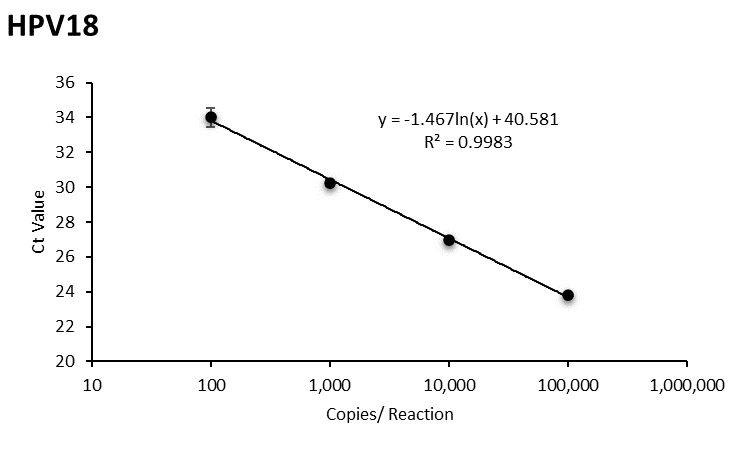 | I) | 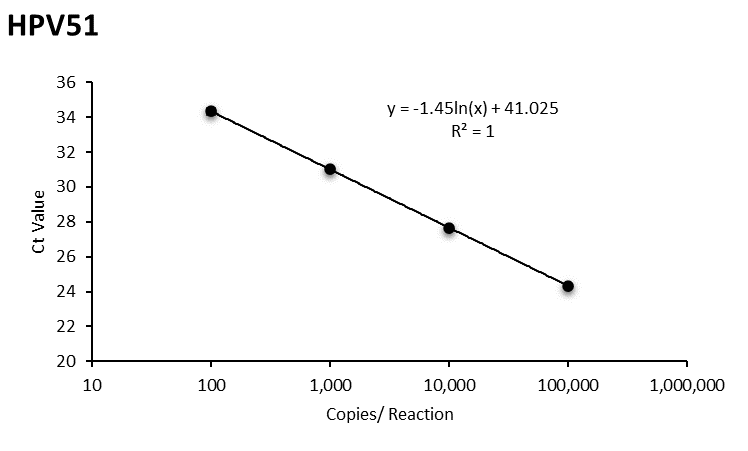 |
| C) | 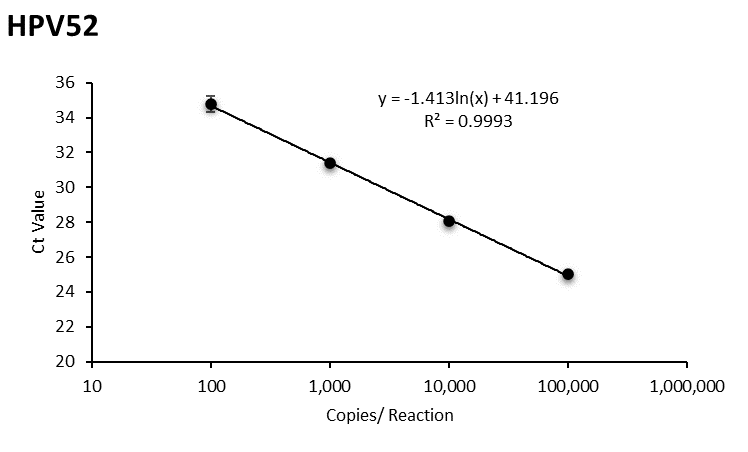 | J) | 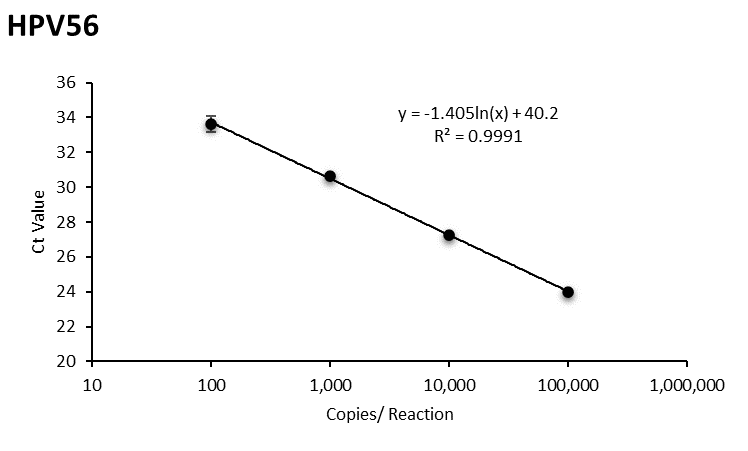 |
| D) | 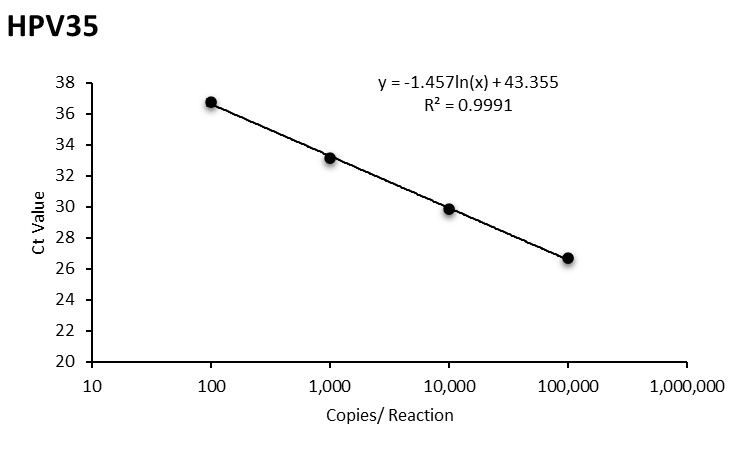 | K) | 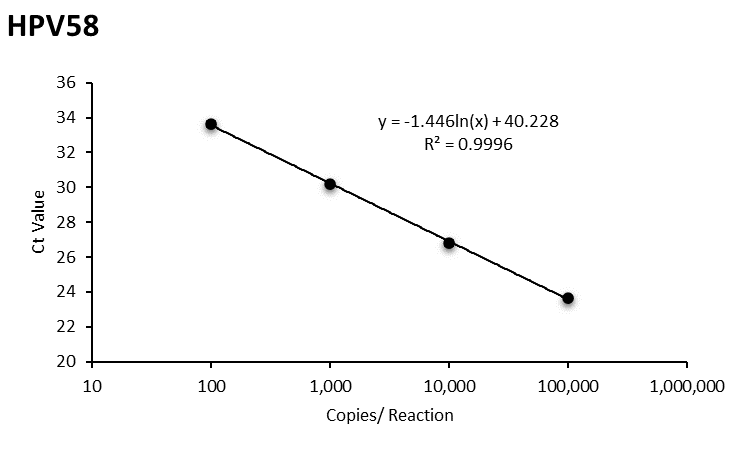 |
| E) | 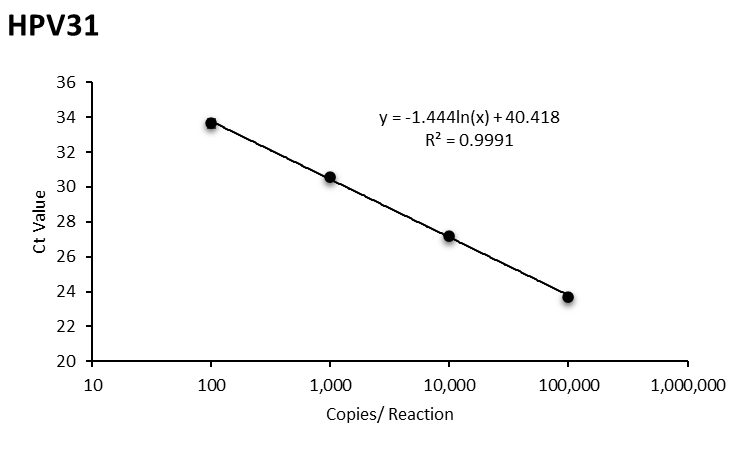 | L) | 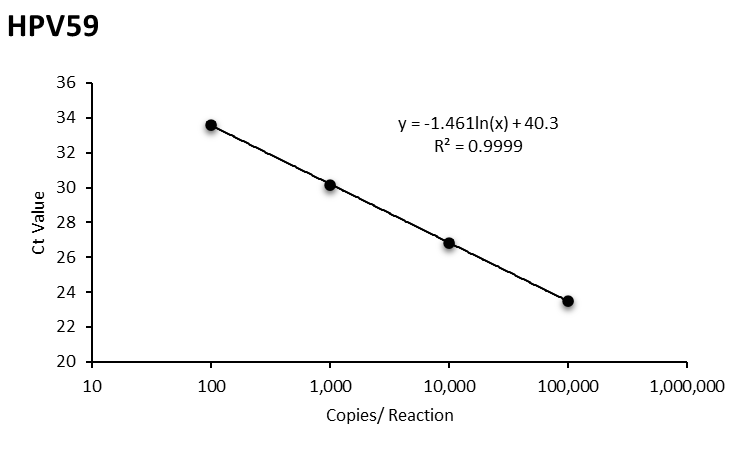 |
| F) | 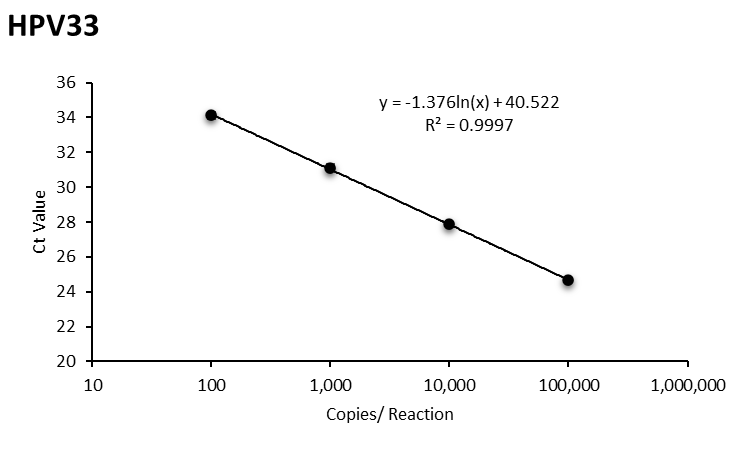 | M) | 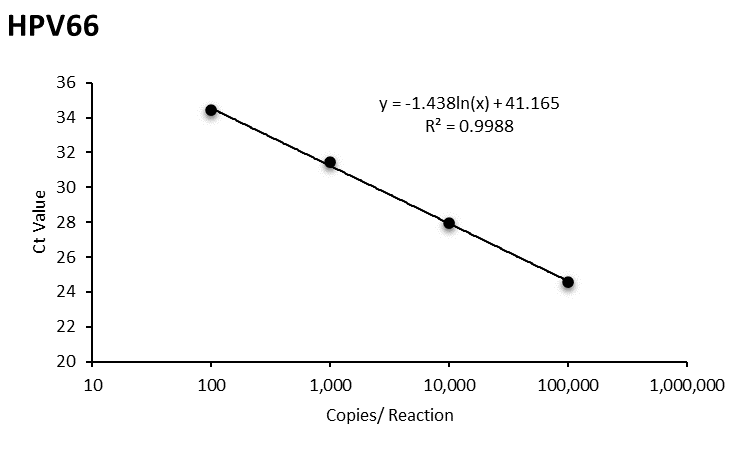 |
| G) | 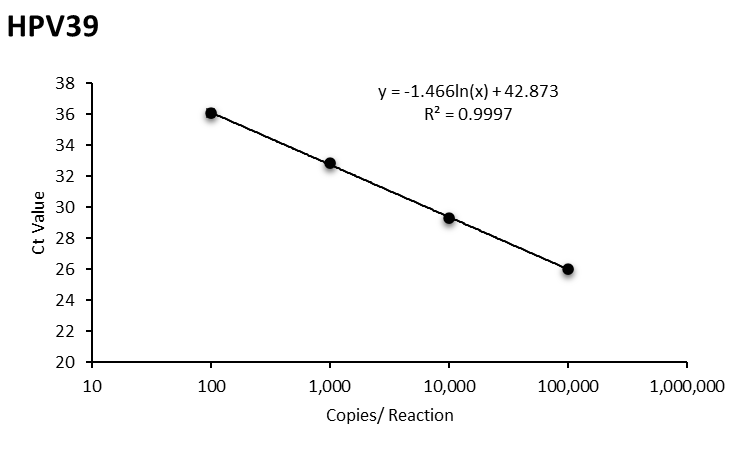 | N) | 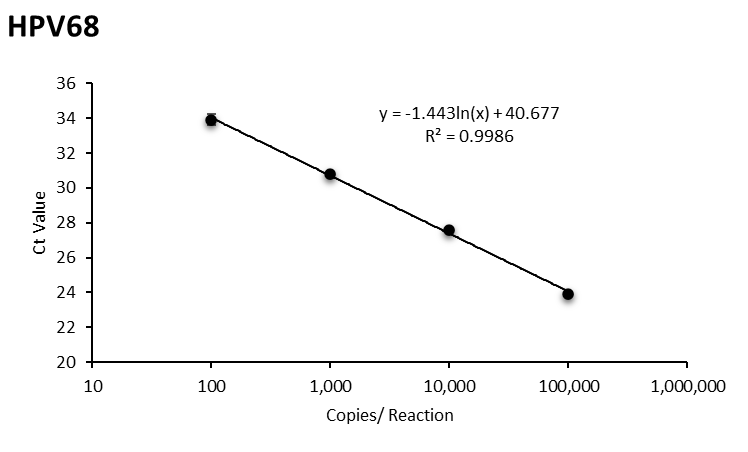 |

Supplementary Fig S1. Linearity assay of ReadyMix in detecting 14 hrHPV types using HPV synthetic DNA as template ranging from 100 to 100,000 GE copies/reaction. A) HPV16; B) HPV18; C) HPV52; D) HPV35; E) HPV31; F) HPV33; G) HPV39; H) HPV45; I) HPV51; J) HPV56; K) HPV58; L) HPV59; M) HPV66; N) HPV68. This assay demonstrated excellent efficiency with a range between 90-110% and R^2^ ~1.


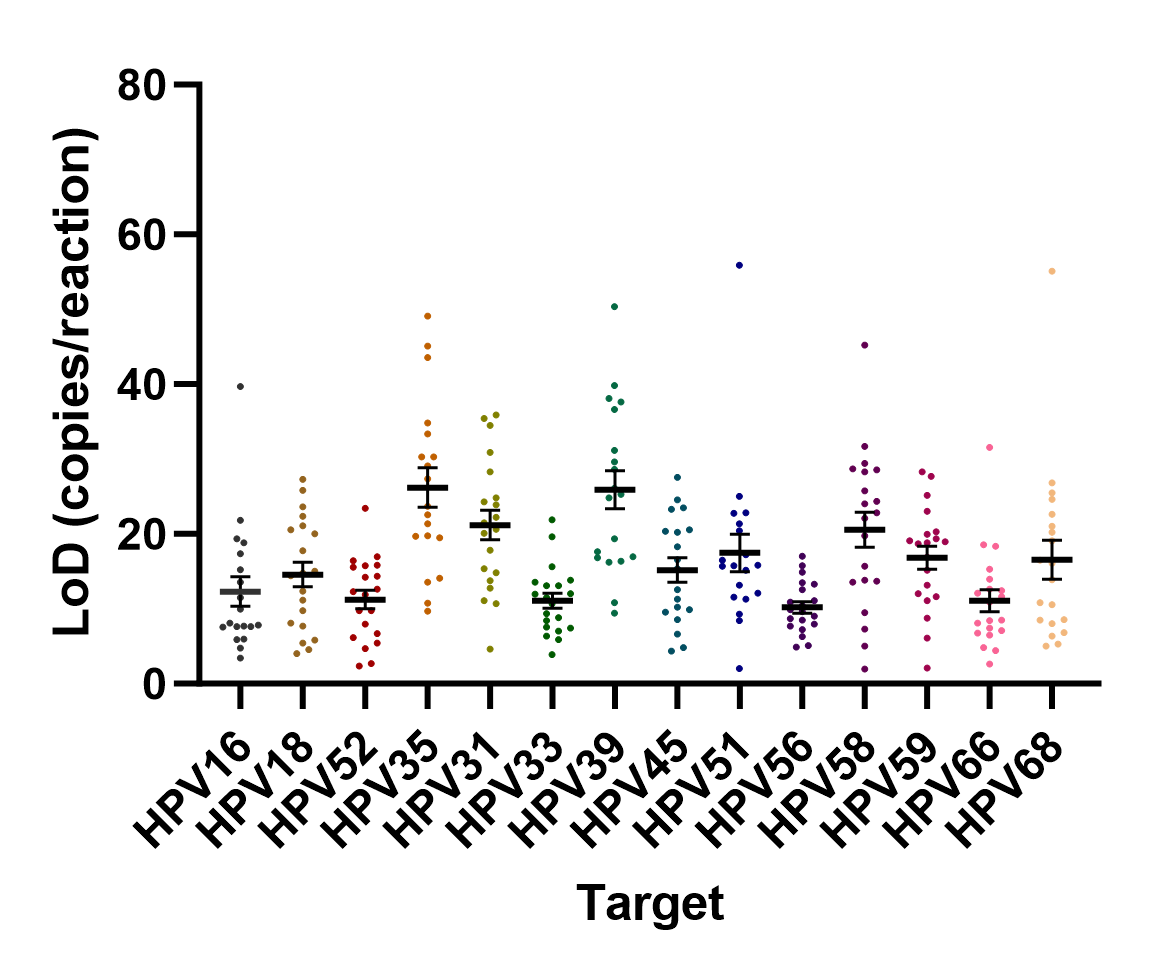


Supplementary Fig S2. Limit of Detection (LoD) of ReadyMix for each hrHPV target ranging from 10-28 copies/20 uL qPCR reaction

Supplementary Table S1. The results of NGS run to investigate the presence of hrHPV in the samples based on E6-E7 amplicon sequencing

| No | Seq ID | Sample Type | False Positive to | | | qPCR Result | hrHPV Detected (%) |
| --- | --- | --- | --- | --- | --- | --- | --- |
|  |  |  | Cervical Swab cobas | Cervical Swab ReadyMix | Urine  cobas |  |  |
| 1 | 140-A-E | Cervical Swab | V | - | - | HPV Other | HPV68 (81.1%), HPV18 (5.7%), HPV51 (4.9%), HPV45 (1.8%), HPV16 (1.8%), HPV82 (1.3%), HPV52 (0.9%), HPV33 (0.5%) |
| 2 | 313-A-E | Cervical Swab | V | - | - | HPV16 | HPV16 (59.3%), HPV52 (20.1%), HPV51 (6.7%), HPV33 (6.1%), HPV31 (3.7%), HPV18 (1.7%), HPV56 (1.3%) |
| 3 | 367-A-E | Cervical Swab | V | - | - | HPV52 | HPV52 (98.5%) |
| 4 | 645-A-E | Cervical Swab | V | - | - | HPV Other | HPV33 (97.6%), HPV16 (1.2%) |
| 5 | 811-A-E | Cervical Swab | V | - | - | HPV Other | HPV56 (57.5%), HPV51 (41.0%) |
| 6 | 904-A-E | Cervical Swab | V | - | - | HPV Other | HPV51 (94.6%), HPV56 (1.6%), HPV52 (1.3%), HPV33 (1.1%) |
| 7 | 140-U-E | Urine | V | - | - | HPV Other | HPV68 (98.6%) |
| 8 | 232-U-E | Urine | - | - | V | HPV Other | HPV59 (74.2%), HPV16 (7.1%), HPV51 (4.2%), HPV58 (3.8%), HPV68 (2.7%), HPV18 (1.5%), HPV31 (1.2%), HPV35 (1.2%), HPV56 (1.1%), HPV45 (0.9%), HPV52 (0.8%) |
| 9 | 313-U-E | Urine | V | - | V | HPV16 | HPV16 (90.6%), HPV58 (2.8%), HPV68 (1.9%), HPV59 (1.9%), HPV33 (1.4%) |
| 10 | 367-U-E | Urine | V | - | - | HPV52, Other | HPV52 (55.7%), HPV56 (44.1%) |
| 11 | 548-U-E | Urine | V | V | - | HPV 52 | HPV52 (91.2%), HPV51 (4.1%), HPV16 (1.7%), HPV18 (1.4%) |
| 12 | 043-U-E | Urine | - | - | - | HPV 18 | HPV18 (93.8%), HPV58 (2.2%), HPV33 (1.9%), HPV31 (1.0%) |
| 13 | 402-U-E | Urine | - | - | - | HPV16 | HPV16 (78.1%), HPV39 (8.0%), HPV52 (4.5%), HPV51 (3.6%), HPV58 (2.7%), HPV33 (1.3%) |
| 14 | 410-U-E | Urine | - | - | - | HPV52 | HPV52 (99.3%) |
| 15 | 413-U-E | Urine | V | V | - | HPV52 | HPV52 (97.1%), HPV18 (0.7%), HPV31 (0.7%) |
| 16 | 191-U-E | Urine | V | V | - | HPV Other | HPV31 (95.0%), HPV51 (1.3%), HPV16 (1.0%), HPV35 (0.5%), HPV56 (0.4%) |
| 17 | 446-U-E | Urine | V | - | - | HPV52 | HPV52 (97.9%), HPV18 (1.0%) |
| 18 | 465-U-E | Urine | - | - | - | HPV52, Other | HPV52 (85.0%), HPV31 (14.1%) |
| 19 | 633-U-E | Urine | - | - | V | HPV Other | HPV51 (97.7%), HPV16 (1.0%) |
| 20 | 645-U-E | Urine | V | - | - | HPV Other | HPV33 (97.9%), HPV51 (0.8%) |
| 21 | 669-U-E | Urine | V | V | - | HPV 18 | HPV18 (94.0%), HPV31 (2.5%), HPV52 (0.9%), HPV16 (0.7%) |
| 22 | 673-U-E | Urine | V | V | - | HPV Other | HPV68 (95.2%), HPV16 (1.9%), HPV18 (1.4%) |
| 23 | 695-U-E | Urine | V | V | V | HPV Other | HPV33 (95.2%), HPV31 (1.9%), HPV52 (0.7%), HPV45 (0.6%) |
| 24 | 710-U-E | Urine | V | V | V | HPV Other | HPV39 (96.2%), HPV52 (1.5%), HPV33 (0.9%) |
| 25 | 718-U-E | Urine | - | V | - | HPV Other | HPV58 (96.7%), HPV51 (1.8%) |
| 26 | 787-U-E | Urine | - | - | V | HPV16, HPV Other | HPV58 (97.5%), HPV16 (1.0%) |
| 27 | 811-U-E | Urine | V | - | - | HPV Other | HPV56 (95.0%), HPV18 (2.1%), HPV51 (1.1%) |
| 28 | 816-U-E | Urine | - | - | - | HPV Other | HPV51 (97.6%), HPV52 (0.6%) |
| 29 | 825-U-E | Urine | V | V | V | HPV52 | HPV52 (98.0%) |
| 30 | 833-U-E | Urine | - | - | - | HPV16 | HPV16 (66.5%), HPV58 (8.5%), HPV18 (6.4%), HPV33 (4.3%), HPV52 (3.2%), HPV45 (3.2%), HPV51 (2.7%), HPV56 (2.7%), HPV68 (2.1%) |
| 31 | 889-U-E | Urine | V | V | - | HPV Other | HPV45 (89.3%), HPV56 (3.6%), HPV51 (3.0%), HPV52 (1.1%), HPV33 (0.5%), HPV18 (0.5%) |
| 32 | 305-A-E | Cervical Swab | N/A | | | HPV16 | HPV16 (52.8%), HPV18 (32.6%), HPV33 (4.9%), HPV51 (3.1%), HPV31 (1.4%), HPV59 (1.4%), HPV56 (1.0%), HPV39 (1.0%) |
| 33 | 616-A-E | Cervical Swab |  |  |  | HPV Other | HPV68 (83.5%), HPV16 (6.9%), HPV52 (3.0%), HPV51 (2.3%), HPV56 (2.1%), HPV82 (1.3%) |
| 34 | 253-A-E | Cervical Swab |  |  |  | HPV52, Other | HPV52 (85.3%), HPV51 (13.2%) |
| 35 | 189-A-E | Cervical Swab |  |  |  | HPV52 | HPV52 (64.7%), HPV82 (33.9%) |
| 36 | 465-A-E | Cervical Swab |  |  |  | HPV Other | HPV31 (91.7%), HPV51 (1.6%), HPV33 (1.4%), HPV56 (1.2%), HPV18 (1.0%), HPV35 (0.6%), HPV52 (0.5%) |
| 37 | 471-A-E | Cervical Swab |  |  |  | HPV52, HPV Other | HPV52 (55.4%), HPV56 (28.4%), HPV51 (14.9%) |
| 38 | 498-A-E | Cervical Swab |  |  |  | HPV18, HPV Other | HPV33 (58.5%), HPV18 (40.6%) |
| 39 | 823-U-E | Urine |  |  |  | HPV16, HPV Other | HPV51 (89.8%), HPV16 (8.5%) |
| 40 | 189-U-E | Urine |  |  |  | HPV52 | HPV52 (99.2%) |
| 41 | CA1-A-E | Cervical Swab |  |  |  | HPV Other | HPV59 (78.5%), HPV18 (4.2%), HPV52 (3.8%), HPV33 (2.8%), HPV82 (2.6%), HPV51 (1.8%), HPV31 (1.4%), HPV58 (1.4%), HPV16 (0.9%), HPV39 (0.9%) |
| 42 | CA1-U-E | Urine |  |  |  | HPV Other | HPV59 (91.2%), HPV33 (4.7%), HPV58 (2.3%) |
| 43 | gBlocks | gBlocks |  |  |  | HPV16, HPV18, HPV52, and HPV Other | HPV18 (20.6%), HPV35 (14.1%), HPV45 (13.3%), HPV58 (10.1%), HPV33 (9.6%), HPV56 (8.7%), HPV51 (7.9%), HPV59 (5.5%), HPV66 (4.2%), HPV52 (2.3%), HPV31 (2.3%), HPV68 (0.88%), HPV16 (0.30%), HPV39 (0.27%). |


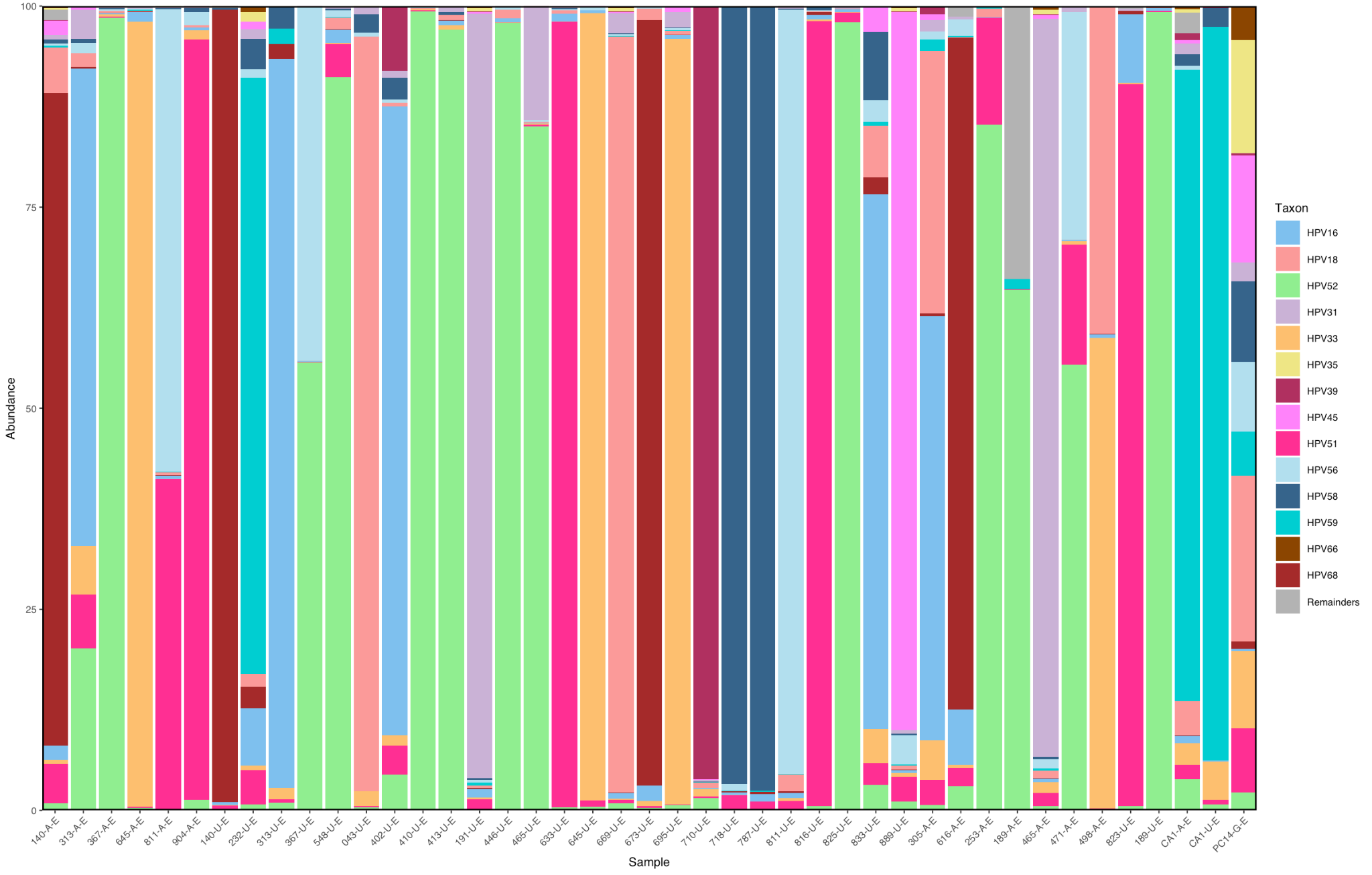


| Output | %>Q30 | Data with > Q30 | Total reads | Reads identified | Number of samples | Average reads/sample |
| --- | --- | --- | --- | --- | --- | --- |
| 0.66 Gb | 83.08% | 0.54 Gb | 1.704.265 | 217.015 | 42 | 5.047 |

Supplementary Fig S3. Stacked taxa bar plot showing the relative abundance of HPV type found in the sample

Supplementary Table S2. HPV detection using cobas in urine versus cervical swab and NGS

| **Urine cobas** | **Cervical Swab cobas & NGS** | | | **k-coefficient** |
| --- | --- | --- | --- | --- |
|  | **Positive** | **Negative** | **Total** |  |
| Positive | 47 | 2 | 49 | 0.84 |
| Negative | 14 | 588 | 602 |  |
| Total | 61 | 590 | 651 |  |
| **Effect size** | **Value (%)** | **95% CI (%)** | |  |
| Sensitivity | 77.05 | 65.09 - 85.81 | |  |
| Specificity | 99.66 | 98.77 - 99.94 | |  |
| Positive Predictive Value | 95.92 | 86.29 - 99.27 | |  |
| Negative Predictive Value | 97.67 | 96.13 - 98.61 | |  |
| Accuracy | 97.54 | 96.04 - 98.48 | |  |

Supplementary Table S3. Demographics of the study population

| **Age Range**  **Average Age** | **(mean** ± SD) | **Total (n)** | **Proportion (%)** | **Prevalence of HPV Type/ Group** | | | | | | | | | | | | | | | | | |
| --- | --- | --- | --- | --- | --- | --- | --- | --- | --- | --- | --- | --- | --- | --- | --- | --- | --- | --- | --- | --- | --- |
|  |  |  |  | **hrHPV ReadyMix** | | | | | | | | | | **cobas^®^ 6800 HPV** | | | | | | | |
|  |  |  |  | **Cervical Swab (n, %)** | | | | | **Urine (n, %)** | | | | | **Cervical Swab (n, %)** | | | | **Urine (n, %)** | | | |
|  |  |  |  | **HPV16** | **HPV18** | **HPV52** | **HPV Others** | **Coinfection** | **HPV16** | **HPV18** | **HPV52** | **HPV Others** | **Coinfection** | **HPV16** | **HPV18** | **HPV Others** | **Coinfection** | **HPV16** | **HPV18** | **HPV Others** | **Coinfection** |
| **20-29** | 26.45 ± 2.46 | 157 | 17.92% | 0 (0) | 0 (0) | 1 (0.64) | 3 (1.91) | 2 (1.27) | 0 (0) | 0 (0) | 2 (1.27) | 2 (1.27) | 2 (1.27) | 0 (0) | 0 (0) | 7 (4.46) | 0 (0) | 0 (0) | 0 (0) | 5 (3.18) | 0 (0) |
| **30-39** | 34.59 ± 2.99 | 346 | 39.50% | 7 (2.02) | 0 (0) | 6 (1.73) | 9 (2.6) | 3 (0.87) | 4 (1.16) | 1 (0.29) | 4 (1.16) | 9 (2.6) | 3 (0.87) | 6 (1.73) | 0 (0) | 15 (4.34) | 2 (0.58) | 5 (1.45) | 2 (0.58) | 16 (4.62) | 1 (0.29) |
| **40-50** | 44.23 ± 3.00 | 373 | 42.58% | 2 (0.54) | 3 (0.8) | 3 (0.8) | 17 (4.56) | 2 (0.54) | 2 (0.54) | 3 (0.8) | 5 (1.34) | 15 (4.02) | 3 (0.8) | 1 (0.27) | 3 (0.8) | 15 (4.02) | 3 (0.8) | 1 (0.27) | 2 (0.54) | 18 (4.83) | 1 (0.27) |
| **Location** |  | | | | | | | | | | | | | | | | | | | | |
| **Jakarta** | N/A | 385 | 43.95% | 3 (0.78) | 2 (0.52) | 6 (1.56) | 11 (2.86) | 1 (0.26) | 2 (0.52) | 2 (0.52) | 7 (1.82) | 9 (2.34) | 0 (0) | 2 (0.52) | 2 (0.52) | 17 (4.42) | 0 (0) | 2 (0.52) | 2 (0.52) | 16 (4.16) | 0 (0) |
| **Bandung** | N/A | 355 | 40.53% | 3 (0.85) | 1 (0.28) | 3 (0.85) | 14 (3.94) | 2 (0.56) | 2 (0.56) | 2 (0.56) | 2 (0.56) | 13 (3.66) | 3 (0.85) | 2 (0.56) | 1 (0.28) | 16 (4.51) | 2 (0.56) | 2 (0.56) | 2 (0.56) | 15 (4.23) | 1 (0.28) |
| **Semarang** | N/A | 136 | 15.53% | 3 (2.21) | 0 (0) | 1 (0.74) | 4 (2.94) | 4 (2.94) | 2 (1.47) | 0 (0) | 2 (1.47) | 4 (2.94) | 5 (3.68) | 3 (2.21) | 0 (0) | 4 (2.94) | 3 (2.21) | 2 (1.47) | 0 (0) | 8 (5.88) | 1 (0.74) |
| **Total** | 37.23 ± 7.28 | 876 | 100.00% | 9 (1.03) | 3 (0.34) | 10 (1.14) | 29 (3.31) | 7 (0.8) | 6 (0.68) | 4 (0.46) | 11 (1.26) | 26 (2.97) | 8 (0.91) | 7 (0.8) | 3 (0.34) | 37 (4.22) | 5 (0.57) | 6 (0.68) | 4 (0.46) | 39 (4.45) | 2 (0.23) |

Supplementary Table S4. HPV16, HPV18, and HPV52 proportion within all HPV cases. Analysis of HPV52 in cobas® 6800 HPV is unavailable due to differences in kit specification

| **Age** | **Total HPV Cases Detected** | | | | **hrHPV ReadyMix** | | | | | | | | **cobas^®^ 6800 HPV** | | | | | |
| --- | --- | --- | --- | --- | --- | --- | --- | --- | --- | --- | --- | --- | --- | --- | --- | --- | --- | --- |
|  | **hrHPV ReadyMix** | | **cobas^®^ 6800 HPV** | | **HPV16** | | **HPV18** | | **HPV52** | | **HPV Other** | | **HPV16** | | **HPV18** | | **HPV Other** | |
|  | **Cervical Swab (n)** | **Urine (n)** | **Cervical Swab (n)** | **Urine (n)** | **Cervical Swab (n, %)** | **Urine (n, %)** | **Cervical Swab (n, %)** | **Urine (n, %)** | **Cervical Swab (n, %)** | **Urine (n, %)** | **Cervical Swab (n, %)** | **Urine (n, %)** | **Cervical Swab (n, %)** | **Urine (n, %)** | **Cervical Swab (n, %)** | **Urine (n, %)** | **Cervical Swab (n, %)** | **Urine (n, %)** |
| **20-29** | 8 | 8 | 7 | 5 | 0 (0) | 0 (0) | 0 (0) | 0 (0) | 3 (37.50) | 4 (50.00) | 5 (62.50) | 4 (50.00) | 0 (0) | 0 (0) | 0 (0) | 0 (0) | 7 (100.00) | 5 (100.00) |
| **30-39** | 29 | 24 | 25 | 25 | 8 (27.59) | 6 (25.00) | 1 (3.45) | 1 (4.17) | 8 (27.59) | 5 (20.83) | 12 (41.38) | 12 (50.00) | 8 (32.00) | 6 (24.00) | 0 (0) | 2 (8.00) | 17 (68.00) | 17 (68.00) |
| **40-50** | 29 | 31 | 25 | 23 | 3 (10.34) | 3 (9.68) | 3 (10.34) | 4 (12.90) | 4 (13.79) | 6 (19.35) | 19 (65.52) | 18 (58.06) | 3 (12.00) | 1 (4.35) | 4 (16.00) | 3 (13.04) | 18 (72.00) | 19 (82.61) |
| **Location** |  | | | | | | | | | | | | | | | | | |
| **Jakarta** | 24 | 20 | 21 | 20 | 3 (12.50) | 2 (10) | 2 (8.33) | 2 (10.00) | 7 (29.17) | 7 (35.00) | 12 (50.00) | 9 (45.00) | 2 (9.52) | 2 (10.00) | 2 (9.52) | 2 (10.00) | 17 (80.95) | 16 (80.00) |
| **Bandung** | 25 | 25 | 23 | 21 | 3 (12.00) | 2 (8.00) | 2 (8.00) | 3 (12.00) | 4 (16.00) | 4 (16.00) | 16 (64.00) | 16 (64.00) | 3 (13.04) | 2 (9.52) | 2 (8.70) | 3 (14.29) | 18 (78.26) | 16 (76.19) |
| **Semarang** | 17 | 18 | 13 | 12 | 5 (29.41) | 5 (27.78) | 0 (0) | 0 (0) | 4 (23.53) | 4 (22.22) | 8 (47.06) | 9 (50.00) | 6 (46.15) | 3 (25.00) | 0 (0) | 0 (0) | 7 (53.85) | 9 (75.00) |
| **Total** | 66 | 63 | 57 | 53 | 11 (16.67) | 9 (14.29) | 4 (6.06) | 5 (7.94) | 15 (22.73) | 15 (23.81) | 36 (54.55) | 34 (53.97) | 11 (19.30) | 7 (13.21) | 4 (7.02) | 5 (9.43) | 42 (73.68) | 41 (77.36) |
